# Leveraging IgG N-glycosylation to infer the causality between T2D and hypertension

**DOI:** 10.1186/s13098-023-01053-6

**Published:** 2023-04-25

**Authors:** Haotian Wang, Yuan Li, Weijie Cao, Jie Zhang, Mingyang Cao, Xiaoni Meng, Di Liu, Youxin Wang

**Affiliations:** 1 Beijing Key Laboratory of Clinical Epidemiology, School of Public Health, Capital Medical University, 10 Xitoutiao, Beijing, 100069 China; 2 Lianyungang Maternal and Child Health Hospital, Lianyungang, 222062 Jiangsu China; 3 Centre for Precision Medicine, Edith Cowan University, Perth, 60127 Australia; 4 Centre for Biomedical Information Technology, Shenzhen Institutes of Advanced Technology, Chinese Academy of Sciences, 1068 Xueyuan Avenue, University Town, Nanshan District, Shenzhen, 518055 Guangdong China

**Keywords:** Bidirectional Mendelian randomization, Glycosylation, IgG N-glycosylation quantitative trait loci, Type 2 diabetes, Hypertension

## Abstract

**Background:**

Observational studies demonstrated a bidirectional association between type 2 diabetes (T2D) and hypertension, whereas Mendelian randomization (MR) analyses supported the causality from T2D to hypertension but not causal from hypertension to T2D. We previously found that IgG N-glycosylation is associated with both T2D and hypertension, and thus IgG N-glycosylation might link the causality between them.

**Methods:**

We carried out a genome-wide association study (GWAS) to identify IgG N-glycosylation-quantitative-trait loci (QTLs) integrating GWAS for T2D and hypertension and then performed bidirectional univariable and multivariable MR analyses to infer the causal association among them. The inverse-variance-weighted (IVW) analysis was performed as the primary analysis, followed by some sensitivity analyses to explore the stability of the results.

**Results:**

Six putatively causal IgG N-glycans for T2D and four for hypertension were identified in the IVW method. Genetically predicted T2D increased the risk of hypertension (odds ratio [OR] = 1.177, 95% confidence interval (95% CI) = 1.037–1.338, *P* = 0.012) and vice versa (OR = 1.391, 95% CI = 1.081–1.790, *P* = 0.010). Multivariable MR showed that T2D remained at risk effect with hypertension ([OR] = 1.229, 95% CI = 1.140–1.325, *P* = 7.817 × 10^–8^) after conditioning on T2D-related IgG-glycans. Conversely, hypertension was associated with higher T2D risk (OR = 1.287, 95% CI = 1.107–1.497, *P* = 0.001) after adjusting for related IgG-glycans. No evidence of horizontal pleiotropy was observed, as MR‒Egger regression provided *P* values for intercept > 0.05.

**Conclusion:**

Our study validated the mutual causality between T2D and hypertension from the perspective of IgG N-glycosylation, further validating the “common soil” hypothesis underlying the pathogenesis of T2D and hypertension.

**Supplementary Information:**

The online version contains supplementary material available at 10.1186/s13098-023-01053-6.

## Highlights

The univariable and multivariable analyses, corrected related IgG-glycans showed that type 2 diabetes was associated with higher hypertension risk and vice versa.

Our findings suggested new insight into the potential mediated effects of IgG N-glycosylation in associations between T2D and hypertension.

**Supplementary Information:**

The online version contains supplementary material available at 10.1186/s13098-023-01053-6.

## Introduction

Type 2 diabetes (T2D) and hypertension, the two leading components of the global burden of disease, commonly coexist [1–4]. This kind of coexistence dramatically increases the risk of cardiovascular disease, stroke, and even death, compared with normotensive and nondiabetic adults [5, 6]. Increasing evidence suggests epidemiological links between T2D and hypertension. In three urban communities in Beijing with 3437 participants, fasting blood glucose and 2-h postprandial blood glucose were independent risk factors for predicting hypertension [7]. In contrast, a cohort of 4.1 million adults reported that 20 mmHg higher systolic blood pressure (SBP) and 10 mmHg higher diastolic blood pressure (DBP) were associated with a 58% and a 52% higher risk of new-onset diabetes [4]. Sun et al*.* performed Mendelian randomization (MR) approach to explore bidirectional associations between T2D and hypertension based on 318,664 UK Biobank participants. They found causal evidence for T2D with hypertension risk, particularly a causal relationship of T2D with SBP, but not from hypertension to T2D [8]. The association between diabetes and hypertension is of continued concern. Understanding the pathological and bidirectional links between T2D and hypertension is of significant public health importance, especially in terms of disease prevention and complication management.

Glycosylation, an important and common post-transcriptional modification, is involved in many biological processes and is also related to disease susceptibility [9, 10]. Immunoglobulin G (IgG), as the main component of antibodies, plays a vital role in the non-specific immune response. Alternative N-glycosylation markedly affects IgG structure and function and, by extension, immune responses, thus acting as a switch between pro- and anti-inflammatory IgG functionality [11, 12]. Meanwhile, epidemiological data revealed the relationship of IgG N-glycosylation with hypertension and T2D. Two large case–control studies found significant associations between T2D and IgG glycan traits involved in pro-inflammatory pathways [13, 14], which may indicate that IgG N-glycosylation plays a functional role in T2D pathophysiology. Similarly, a multiple ethnic cross-sectional study showed that IgG N-glycosylation contributed to the pathogenesis of hypertension via its effect on pro- and/or anti-inflammatory pathways [15]. The above findings suggested new insight into the potential mediated effects of IgG N-glycosylation in associations between T2D and hypertension.

IgG glycans are highly responsive to genetic stimuli and frequently change in response to various pathophysiological conditions. IgG N-glycosylation quantitative trait loci (IgG N-glycan-QTLs), which act as intermediate phenotypes, might afford greater power to detect the association between T2D and hypertension. Previous studies incorporating QTL information (e.g., protein levels) into GWAS analyses indicated that QTL variants provide excellent instrumental variables (IVs) for exposure in MR analysis. This direction of inquiry can be extended to other ‘‘-omic’’ data types to gain further insights into the mechanistic pathway between genetic variants and causally associated traits [16, 17]. Our recent study showed that identifying IgG N-glycan-QTLs variants and then linking them to rheumatoid arthritis-associated genetic variants from GWAS confirmed causal associations and might pinpoint the molecular mechanisms underlying genetic susceptibility to rheumatoid arthritis [18]. As mentioned above, the new features might be revealed in bidirectional causality between T2D and hypertension using IgG N-glycan-QTLs.

MR, a form of IV analysis that leverages the random assortment of genetic variants during gamete formation and minimizes the influence of confounding as well as reverse causation, issues of observational studies, has been popularly used in estimating causal inference between exposures and outcomes [19–21]. Multivariable Mendelian randomization is an extension of univariable MR that takes separate but correlated exposures into account [22, 23]. Multivariable MR aimed to disentangle the direct effect for each exposure not mediated by other correlated exposures for a range of health outcomes. If causal associations are observed in multivariable MR, the conclusion can be drawn that the exposure has direct associations with the outcome that does not work via the mediator. Therefore, with the use of integrating IgG N-glycan-QTLs and GWAS for T2D and hypertension data, we performed bidirectional univariable and multivariable MR analyses to explore the causal association between hypertension and T2D from the perspective of IgG N-glycosylation.

## Materials and methods

### Study design

**Fig. 1:**
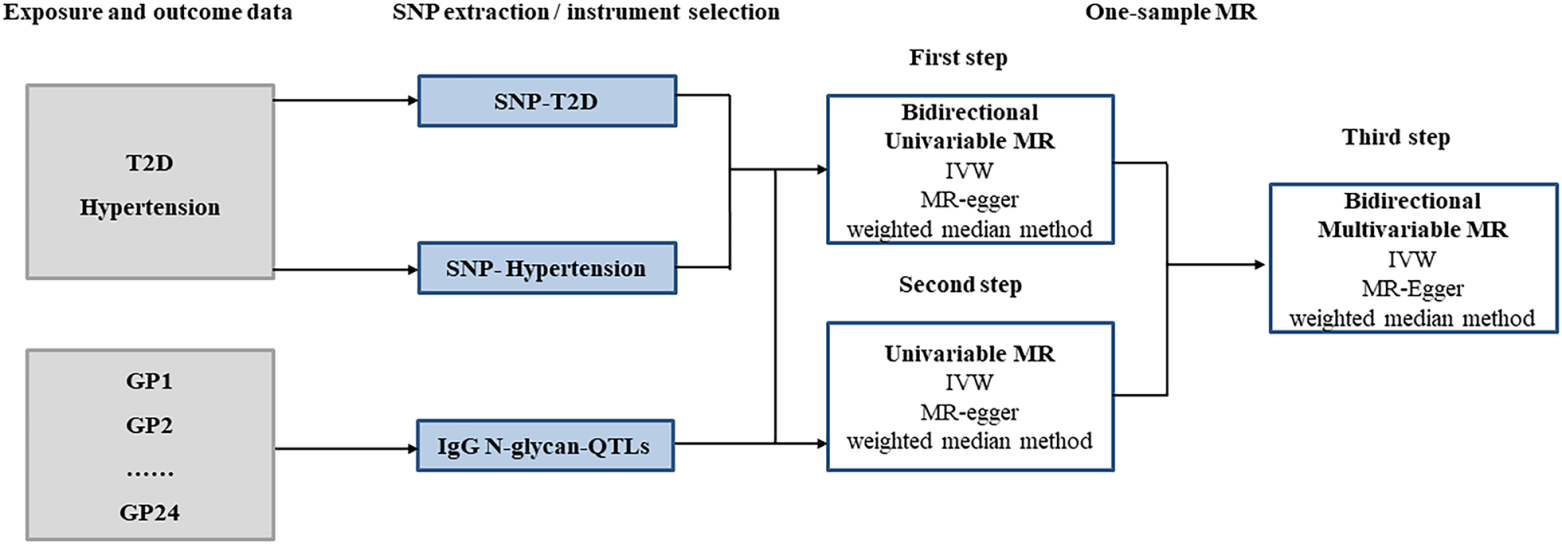
Study overview and Mendelian randomization analysis. GP: glycan peak; IVW: inverse-variance-weighted; MR: Mendelian randomization; SNP: single nucleotide polymorphism; T2D: Type 2 diabetes

Our study is reported according to the Strengthening the Reporting of Observational Studies in Epidemiology (STROBE) guideline [24]. We used a one-sample bidirectional MR design to infer the complex causal associations among IgG N-glycosylation, T2D, and hypertension. There are three assumptions followed in MR analysis [25]: (1) a strong association between genetic variants and exposure; (2) no association with confounders of the exposure-outcome association; and (3) no effect of variants on outcome unless via exposure. Figure 1 illustrated our study overview and MR analyses. It consists of 3 steps described below. First, we adopted univariable MR analyses to assess bidirectional causal references between T2D and hypertension. In the second step, we performed univariable MR analyses with IgG N-glycans as the exposure, T2D or hypertension as the outcome respectively, and the relevant IgG N-glycan-QTL variants as the IVs. The identified causal IgG N-glycans with T2D or hypertension were used for further multivariable MR analyses. The third step, multivariable MR analysis, is an extension of MR analysis that takes the IgG N-glycan association on T2D or hypertension into account for exploring bidirectional associations between T2D and hypertension. With the use of multivariable MR analysis, we calculated the direct effect that contributed to the exposure of interest and adjusted for potential confounders.

### Study participants

In the present study, data were collected from a community-based cohort, cross-sectional study from Xuanwu Hospital in Beijing (from September 2009 to September 2012 of the baseline population). The detailed study design and assessment methods were described in a previous study [15, 26]. Written informed consent was obtained from each subject at the beginning of the study, and the study was approved by the Ethics Committee of the Capital Medical University, Beijing, China. Ethics approval was given in compliance with the Declaration of Helsinki. In addition, we carried out the two-sample MR design as replication analyses by utilizing GWAS summary statistics from Biobank Japan (http://jenger.riken.jp/en/result#). Since GWAS summary data of hypertension was not included in Biobank Japan, the analysis was based on GWAS summary data of T2D and GWAS summary data of SBP [27, 28]. The ethical approval statement was not sought, as we obtained summary-level data from open publicly available datasets.

### Data collection

All participants from Xuanwu Hospital in Beijing were required to undergo a physical examination that included anthropometric and biochemical measurements, as delineated in a previous study. Demographic characteristics of participants, including age, gender, and ethnicity, were collected by a questionnaire. SBP and DBP were measured three times on the right arm in a day with a standard mercury sphygmomanometer, and subjects were required to rest for at least 5 min for each measurement. The participants were then classified into the hypertension group (mean SBP ≥ 140 mmHg or mean DBP ≥ 90 mmHg) or the normal blood pressure group (mean SBP < 120 mmHg and mean DBP < 80 mmHg) [3]. The fasting blood glucose (FBG) concentrations were measured by the glucose oxidase–peroxidase method (Mind Bioengineering Co. Ltd., Shanghai, China). The diagnosis of T2D was made by physicians according to the 1999 WHO Criteria (FBG greater than or equal to 7.0 mmol/L) [29].

After overnight fasting, two tubes of blood (5 mL) were collected in the morning by venipuncture. One sample was taken in vacuum negative pressure tubes not containing ethylene diamine tetraaceticethylenediaminetetraacetic acid (EDTA) to acquire serum (2 mL), which was used to detect the blood biochemistry indexes, and the other sample was taken in vacuum negative pressure tubes containing EDTA. The whole blood was centrifuged at 3000 rpm for 10 min, then the plasma (3 mL) was separated, which was used to measure IgG N-glycosylation, and the blood cells (2 mL) were separated, which was used to detect genetic variants. All collected blood samples were processed within 8 h and stored at − 80 °C until further measurement.

### Genotyping and genotype imputation

The genotyping procedures were conducted with Illumina Omni Zhonghua chips (Illumina, San Diego, CA, USA). Quality control was conducted as described previously [14]. Genotypes were imputed from the 1000 Genomes Project panel phase 3 based on the East Asian population using the Michigan Imputation Server. SNPs with minor allele frequency (MAF) > 0.01 and imputation quality ratio > 0.3 were retained, yielding 7,108,659 imputed SNPs that were used for further IgG N-glycan-QTL mapping. Based on not facing the problem of population stratification, our main analyses did not correct the principal component. We performed GWAS adjusting for the top three principal components as sensitivity analyses.

### IgG N-glycosylation

IgG N-glycan profile analysis was performed by the method of hydrophilic interaction chromatography-ultraperformance liquid chromatography. The protocol of the method was reported as described in detail previously [30]. Finally, 24 glycan peaks (GPs, GP1-GP24) were used for further IgG N-glycan-QTL mapping. The structures of glycans in each peak were reported as described in detail previously [30]. To control the experimental variability, we adopted normalization methods and batch correction to process the glycan data so that all samples were comparable.

### Selection of the instrumental variable

IgG N-glycan-QTL analysis was performed to select IVs for 24 IgG N-glycans. Briefly, linear regression, adjusted for age and sex, was conducted to test the association between each SNP and IgG N-glycans, with each IgG N-glycan as the dependent variable of interest and SNP as the independent variable. In addition, GWASs of T2D and hypertension were performed after adjusting for the effect of the same confounders, including age and sex. A relatively conservative Bonferroni correction was used (i.e., *P* < 1 × 10^−5^). Since various IgG N-glycans are highly correlated and the mechanism regulating IgG N-glycosylation is not specific [31, 32], we have not ruled out IgG N-glycan-QTLs overlapping between GPs. However, as many significant IgG N-glycan-QTLs are in high linkage disequilibrium (LD), we retained the IgG N-glycan-QTLs at LD r^2^ < 0.001. The LD proxies were defined using 1000 genomes of East Asian samples [33]. Then, these IgG N-glycan-QTLs corresponding to the causal IgG N-glycans for T2D or hypertension were used as IVs for T2D or hypertension. For the replication analysis, we selected SNPs as IVs that were associated with the T2D and SBP significantly (*P* < 5 × 10^−8^) and independently (not in LD r^2^ < 0.001 and distance > 10,000 kb) with the other SNPs.

### Statistical analysis

We conducted a one-sample MR study with GWAS data from Xuanwu hospital and a two-sample MR study leveraging GWAS data from BBJ Japan to estimate the causal associations. The univariable MR evaluated causal associations in T2D and hypertension, while multivariable MR corrected other effects of IgG-glycans in the model to obtain independent direct effects of T2D and hypertension. MR analysis was undertaken by inverse-variance weighted (IVW) regression. The heterogeneity between SNPs was estimated by Cochran Q statistic. A random-effects IVW model was used if heterogeneity existed; otherwise, a fixed-effects IVW model was performed. We also conducted weighted median and MR‒Egger analyses to test the robustness of the results. The MR‒Egger method was used to assess the robustness of estimates to potential violations of the standard IVs assumptions attributed to directional pleiotropy. For individual-level data, the two-stage least squares (2SLS) method was also applied to estimate the causality between IgG N-glycans, T2D, and hypertension[34]. In the first stage, the exposure was regressed on the genetic variants using logistic regression or linear models to generate predicted values of exposure. In the second stage, the outcomes were regressed on the predicted exposure adjusted for age and sex in the logistic model. In terms of various estimates for different measures, we preferred to report the results of the IVW method. The results were presented as odds ratios (OR) with their 95% confidence interval (CI) and beta with standard error (SE) of outcomes per genetically predicted increase in each exposure factor.

Data cleaning and statistical analysis were performed by the “MendelianRandomization” and “TwosampleMR” packages using R version 4.1.2 and PLINK 1.9. *P* < 0.05 was considered as suggestive of evidence for a potential association.

## Results

### Participant characteristics

The average age of the 536 participants included was 47.87 years, and 31.53% were males (Additional file 5: Table S1). There were 62 (11.57%) and 164 (30.59%) participants with T2D and hypertension, respectively. Of note, 58.06% of T2D patients had hypertension, while 21.95% of hypertensive participants were T2D. Hypertension was 2.15 times more frequent in patients with diabetes compared with those who did not have diabetes. Moreover, T2D was 3.14 times more frequent in patients with hypertension than in those without hypertension.

### Estimated causal association of IgG N-glycosylation and T2D with hypertension

We performed a univariable MR to evaluate the causal associations between T2D and hypertension. We used the causal SNPs that were associated with T2D as IVs (N_SNPs_ = 6), as shown in Additional file 5: Table S2. As shown in Table 1, genetically predicted T2D showed an increased risk of hypertension (OR (95% CI) = 1.177 (1.037–1.338), *P* = 0.012). The results of the 2SLS method of IV analysis were reported in Additional file 5: Table S17 and the causal association of T2D with hypertension was marginally significant (OR = 1.334, 95% CI = 0.967–1.840; *P* = 0.080). The effect of T2D on hypertension via univariable MR analysis was consistent in the sensitivity analysis (OR = 1.199, 95% CI = 1.093–1.316, *P* = 1.301 × 10^–4^). While the results were not consistent in the MR‒Egger (OR (95% CI) = 1.548 (0.960–2.494, *P* = 0.073) and weighted median method (OR (95% CI) = 1.142 (0.941–1.387), *P* = 0.180), we preferred the IVW method for more precise results as the main method. There is no proof of directional pleiotropy for associations of T2D with hypertension in MR–Egger regression (*P*s for intercept > 0.05).

**Table 1:** Causal effect estimates on hypertension via Univariable Mendelian randomization

Exposures	SNP (N)	OR (95%CI) (*P* value)	MR‒Egger (*P* value)	Weighted median method (*P* value)	MR‒Egger intercept (*P* value)
**T2D**	**6**	**1.177 (1.037–1.338) (0.012)**	1.548 (0.960–2.494) (0.073)	1.142 (0.941–1.387) (0.180)	− 0.384 (0.289)
GP1	167	0.976 (0.914–1.042) (0.465)	1.054 (0.907–1.224) (0.493)	1.032 (0.929–1.146) (0.554)	− 0.061 (0.327)
GP2	39	1.113 (0.956–1.297) (0.169)	**1.340 (1.005–1.789) (0.046)**	1.091 (0.877–1.358) (0.434)	− 0.121 (0.141)
GP3	38	1.013 (0.851–1.206) (0.887)	1.114 (0.789–1.573) (0.539)	1.146 (0.902–1.456) (0.265)	− 0.097 (0.336)
GP4	32	1.101 (0.903–1.343) (0.341)	1.290 (0.871–1.911) (0.204)	1.064 (0.806–1.406) (0.661)	− 0.095 (0.382)
GP5	77	0.987 (0.888–1.096) (0.801)	1.208 (0.969–1.507) (0.094)	1.070 (0.912–1.256) (0.408)	− **0.150 (0.048)**
GP6	25	0.954 (0.787–1.158) (0.635)	0.751 (0.479–0.177) (0.212)	0.960 (0.689–1.339) (0.812)	0.138 (0.198)
GP7	29	1.091 (0.925–1.287) (0.302)	1.284 (0.913–1.804) (0.150)	1.101 (0.861–1.408) (0.442)	− 0.109 (0.260)
GP8	16	0.941 (0.709–1.250) (0.675)	0.920 (0.518–1.633) (0.776)	0.832 (0.602–1.151) (0.267)	0.015 (0.897)
GP9	15	1.210 (0.861–1.699) (0.272)	1.037 (0.383–2.809) (0.944)	1.286 (0.859–1.926) (0.222)	0.063 (0.719)
GP10	25	0.910 (0.745–1.112) (0.356)	0.712 (0.458–1.105) (0.130)	0.965 (0.729–1.279) (0.806)	0.125 (0.298)
GP11	18	0.920 (0.732–1.155) (0.472)	0.913 (0.538–1.551) (0.737)	0.958 (0.683–1.345) (0.806)	− 0.008 (0.941)
GP12	46	0.930 (0.808–1.071) (0.316)	1.151 (0.876–1.514) (0.312)	0.934 (0.758–1.150) (0.518)	− 0.126 (0.106)
GP13	58	0.970 (0.858–1.096) (0.621)	0.987 (0.776–1.254) (0.912)	0.998 (0.827–1.203) (0.979)	0.008 (0.910)
GP14	18	0.867 (0.656–1.145) (0.314)	1.104 (0.482–2.528) (0.815)	0.774 (0.514–1.168) (0.223)	− 0.102 (0.525)
**GP15**	**18**	**0.775 (0.624–0.964) (0.022)**	1.128 (0.626–2.035) (0.689)	0.813 (0.774–1.324) (0.197)	− 0.184 (0.170)
GP16	26	1.038 (0.868–1.241) (0.687)	0.759 (0.461–1.250) (0.278)	1.012 (0.774–1.324) (0.930)	0.142 (0.164)
**GP17**	**60**	**0.828 (0.734–0.934) (0.002)**	1.091 (0.855–1.392) (0.483)	0.884 (0.734–1.064) (0.191)	− **0.162 (0.034)**
GP18	12	1.099 (0.815–1.482) (0.537)	1.271 (0.551–2.934) (0.574)	1.165 (0.755–1.798) (0.490)	− 0.059 (0.751)
GP19	23	0.957 (0.793–1.154) (0.645)	1.040 (0.679–1.592) (0.857)	0.946 (0.712–1.258) (0.704)	− 0.083 (0.401)
**GP20**	**78**	**0.833 (0.751–0.923) (5.155 × 10**^**–4**^**)**	0.941 (0.725–1.223) (0.650)	**0.845 (0.720–0.992) (0.040)**	− 0.112 (0.283)
GP21	36	1.061 (0.910–1.236) (0.450)	1.351 (0.967–1.887) (0.078)	1.065 (0.848–1.338) (0.588)	− 0.145 (0.122)
**GP22**	**259**	**0.903 (0.857–0.952) (1.690 × 10**^**–4**^**)**	1.017 (0.856–1.207) (0.852)	0.970 (0.892–1.055) (0.481)	− 0.105 (0.238)
GP23	19	1.121 (0.883–1.424) (0.347)	1.599 (0.946–2.702) (0.080)	1.300 (0.941–1.796) (0.111)	− 0.176 (0.137)
GP24	21	1.148 (0.899–1.466) (0.270)	1.718 (0.898–3.286) (0.102)	1.062 (0.778–1.451) (0.703)	− 0.154 (0.212)

The results in bold indicate that the p value is less than 0.05

CI: confidence intervals; GP: glycan peak; OR: odds ratio; SE: standard error; SNP: single nucleotide polymorphism; T2D: Type 2 diabetes

The identified IgG N-glycan-QTLs as IVs for each IgG N-glycan trait were strongly associated with IgG N-glycosylation, and detailed information of select IgG N-glycan-QTLs was presented in Additional file 5: Table S3. Six IgG N-glycans, including GP2, GP5-6, GP15, GP22, and GP24, presented causal relationships for the outcome of T2D via univariable MR analyses (Table 2). The findings of GP5 and GP22 from replication analyses were consistent with the primary analysis (OR = 1.013, 95% CI = 1.004–1.023, *P* = 0.005; OR = 0.995, 95 CI% = 0.991–1.000; *P* = 0.046; Additional file 5: Table S11). Of note, the MR‒Egger and weighted median methods validated the association of GP2, GP6, and GP22 with T2D. In addition, the MR‒Egger intercept analysis provided evidence for horizontal pleiotropy in GP5, GP13, GP17, and GP22.

**Table 2:** Causal effect estimates on diabetes via univariable Mendelian randomization

Exposures	SNP (N)	OR (95%CI) (*P* value)	MR‒Egger (*P* value)	Weighted median method (*P* value)	MR‒Egger intercept (*P* value)
**Hypertension**	**6**	**1.391 (1.081–1.790) (0.010)**	2.018 (0.879–4.637) (0.098)	1.132 (0.928–1.381) (0.222)	− 0.350 (0.342)
GP1	134	1.074 (0.952–1.213) (0.247)	1.262 (0.985–1.618) (0.066)	1.112 (0.943–1.311) (0.206)	− 0.122 (0.204)
**GP2**	**37**	**1.435 (1.190–1.731) (1.550 × 10**^**–4**^**)**	**1.567 (1.042–2.356) (0.031)**	**1.610 (1.174–2.207) (0.003)**	− 0.057 (0.635)
GP3	35	1.169 (0.932–1.466) (0.177)	**1.618 (1.023–2.557) (0.039)**	**1.509 (1.072–2.124) (0.018)**	− 0.200 (0.142)
GP4	29	1.219 (0.937–1.586) (0.140)	1.043 (0.584–0.1863) (0.887)	1.271 (0.848–1.903) (0.246)	0.100 (0.518)
**GP5**	**62**	**0.819 (0.681–0.984) (0.033)**	1.394 (0.960–2.024) (0.081)	0.936 (0715–1.227) (0.634)	− **0.317 (0.008)**
**GP6**	**24**	**1.498 (1.515–1.950) (0.003)**	**2.633 (1.506–4.605) (0.001)**	**1.740 (1.115–2.716) (0.015)**	− 0.285 (0.056)
GP7	27	0.892 (0.655–1.214) (0.466)	1.590 (0.921–2.744) (0.096)	0.935 (0.629–1.392) (0.742)	0.282 (0.060)
GP8	15	0.787 (0.546–1.133) (0.197)	0.748 (0.285–1.964) (0.556)	0.808 (0.480–1.359) (0.422)	0.008 (0.965)
GP9	15	1.069 (0.618–1.848) (0.811)	1.546 (0.543–4.403) (0.415)	0.917 (0.548–1.536) (0.743)	− 0.124 (0.507)
GP10	23	0.743 (0.526–1.050) (0.092)	1.257 (0.637–2.480) (0.510)	0.704 (0.455–1.091) (0.117)	− 0.256 (0.154)
GP11	17	0.936 (0.676–1.298) (0.693)	1.015 (0.476–2.165) (0.970)	0.944 (0.571–1.561) (0.823)	− 0.035 (0.828)
GP12	41	0.851 (0.659–1.099) (0.216)	1.216 (0.776–1.904) (0.393)	0.996 (0.711–1.395) (0.980)	− 0.184 (0.127)
GP13	48	0.916 (0.725–1.157) (0.462)	1.462 (0.975–2.193) (0.066)	1.010 (0.746–1.367) (0.948)	**0.230 (0.030)**
GP14	18	0.686 (0.424–1.110) (0.125)	0.812 (0.273–2.413) (0.707)	0.678 (0.369–1.246) (0.211)	− 0.064 (0.767)
**GP15**	**17**	**0.560 (0.384–0.816) (0.003)**	1.313 (0.436–3.953) (0.628)	0.602 (0.357–1.016) (0.057)	− 0.349 (0.153)
GP16	25	1.124 (0.860–1.468) (0.394)	1.138 (0.530–2.447) (0.740)	1.148 (0.777–1.697) (0.488)	− 0.007 (0.962)
GP17	53	0.889 (0.727–1.087) (0.251)	**1.517 (1.044–2.204) (0.029)**	1.014 (0.754–1.363) (0.927)	− **0.291 (0.011)**
GP18	12	1.095 (0.634–1.892) (0.745)	1.496 (0.464–4.818) (1.496)	1.423 (0.757–2.674) (0.273)	− 0.156 (0.551)
GP19	22	1.286(0.984–1.681) (0.066)	1.303 (0.717–2.368) (0.385)	1.405 (0.931–2.122) (0.105)	− 0.017 (0.901)
GP20	62	0.857 (0.719–1.022) (0.086)	0.964 (0.617–1.504) (0.870)	0.891 (0.691–1.149) (0.374)	− 0.057 (0.731)
GP21	34	0.937 (0.725–1.212) (0.621)	1.204 (0.721–2.011) (0.478)	0.988 (0.692–1.411) (0.948)	− 0.202 (0.153)
**GP22**	**219**	**1.178 (1.176–1.290) (4.097 × 10**^**–4**^**)**	**1.662 (1.270–2.174) (2.134 × 10**^**–4**^**)**	**1.253 (1.104–1.422) (4.948 × 10**^**–4**^**)**	**0.342 (0.013)**
GP23	19	1.042 (0.704–1.542) (0.837)	0.921 (0.427–1.984) (0.833)	1.074 (0.683–1.689) (0.756)	0.084 (0.623)
**GP24**	**21**	**1.925 (1.369–2.706) (1.661 × 10**^**–4**^**)**	1.532 (0.539–4.354) (0.424)	**2.182 (1.361–3.498) (0.001)**	0.090 (0.650)

The results in bold indicate that the *P* value is less than 0.05

CI: confidence intervals; GP: glycan peak; OR: odds ratio; SE: standard error; SNP: single nucleotide polymorphism

**Fig. 2:**
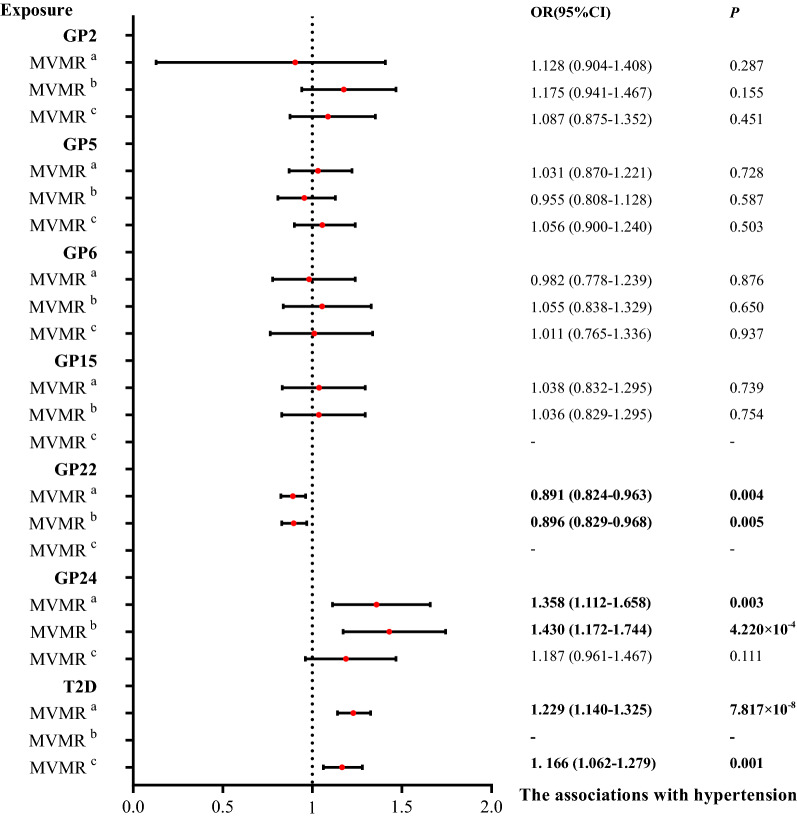
Causal effect estimates on hypertension via Multivariable Mendelian randomization. The results of significant IgG N-glycans and T2D with hypertension are marked “a” in the top right corner, while the results of only IgG N-glycans are marked “b”, and the results for removing overlapping IgG N-glycans are marked “c”. GP: glycan peak; T2D: Type 2 diabetes

Considering that univariable MR analysis showed that IgG N-glycans influenced hypertension and T2D, it is necessary to correct the causal reference of IgG N-glycans between T2D and hypertension. We performed a multivariable MR to simultaneously estimate the direct effect of selected IgG N-glycans and T2D on hypertension (N_SNPs_ = 305). The causal associations for T2D on hypertension were statistically significant (OR = 1.229, 95% CI = 1.140–1.325, *P* = 7.817 × 10^–8^; Fig. 2, Additional file 5: Table S5), which was validated by the MR‒Egger method (OR = 1.226, 95% CI = 1.137–1.322, *P* = 1.312 × 10^–7^, Additional file 5: Table S5, Additional file 1: Fig. S1), the weighted median method (OR = 1.231, 95% CI = 1.119–1.354, *P* = 1.920 × 10^–5^, Additional file 2: Fig. S2). The sensitivity analyses showed robust findings in the multivariable MR model (OR = 1.179, 95% CI = 1.095–1.269, *P* = 1.185 × 10^–5^). For the replication analysis, there was evidence to support a protective association between T2D and SBP (OR = 0.960, 95% CI = 0.784–1.174, *P* = 0.017; Additional file 5: Table S15). GP24 presented an increased risk of hypertension (OR = 1.358, 95% CI = 1.112–1.658, OR = 1.361, 95% CI = 1.114–1.661; OR = 1.412, 95% CI = 1.065–1.870; all *P*s < 0.05). We observed that GP22 had a protective association with T2D by the IVW and MR‒Egger methods. Then, we only adopted these selected IgG N-glycans as exposures in multivariable MR analysis, and GP24 and GP22 yielded similar results in IVW methods (OR = 0.896, 95% CI = 0.829–0.968),* P* = 0.005; OR = 1.430, 95% CI = 1.172–1.744, *P* = 4.220 × 10^–4^). On account of two IgG N-glycans, GP15, and GP22, overlapped in the univariable MR of T2D and hypertension, we examined the association of genetically proxied T2D on hypertension after removing those overlapping IgG N-glycans. All methods showed evidence of genetically predicted increased risk between T2D and hypertension (OR = 1.166, 95% CI = 1.062–1.279, OR = 1.141, 95% CI = 1.037–1.255; OR = 1.160, 95% CI = 1.021–1.319, all *P*s < 0.05). No evidence of horizontal pleiotropy for associations on the abovementioned multivariable MR analyses was observed, as MR–Egger regression provided intercept for *P* values for intercept > 0.05 (*P* = 0.560, *P* = 0.309,* P* = 0.061, respectively; Additional file 5: Table S5). The sensitivity analyses for associations of IgG N-glycans on hypertension were presented on Additional file 5: Tables S7 and S9.

### Estimated causal association of IgG N-glycosylation and hypertension with T2D

In the other direction, with genetic liability for hypertension as an exposure (Table 1), we performed univariable MR to explore the causal association on the outcome of T2D. Six related IgG N-glycan-QTL SNPs were used in backward MR analysis (Additional file 5: Table S2). The IVW method determined that hypertension was associated with higher T2D risk (OR (95% CI = 1.391 (1.081–1.790), *P* = 0.010; Table 2). The IV analysis of individual-level data (2SLS method) found positive but not significant evidence that hypertension was associated with T2D (OR = 1.169, 95% CI = 0.911–1.500, *P* = 0.221; Additional file 5: Table S18). The weighted median method (OR (95% CI = 1.132 (0.928–1.381), *P* = 0.222) and MR‒Egger method (OR (95% CI = 2.018 (0.879–4.637), *P* = 0.098) were inconsistent with the IVW method, and their difference may be due that IVW has more power in causal reference than others. The MR–Egger regression reported no evidence of horizontal pleiotropy as its intercept *P* value > 0.05.

Additional file 5: Table S4 provided descriptive information on selected IgG N-glycan-QTL SNPs. Four IgG N-glycans, GP15, GP17, GP20, and GP22, were associated with hypertension via univariable MR analyses. Four IgG N-glycans, GP15, GP17, GP20, and GP22, were associated with hypertension via univariable MR analyses, but this was not confirmed in the replication analysis (Additional file 5: Table S12). Of note, the results of GP20 using the weighted median method and 2SLS regression analyses were consistent with the primary analysis. MR–Egger intercept analysis indicated some evidence of horizontal pleiotropy for the association of GP17.

**Fig. 3:**
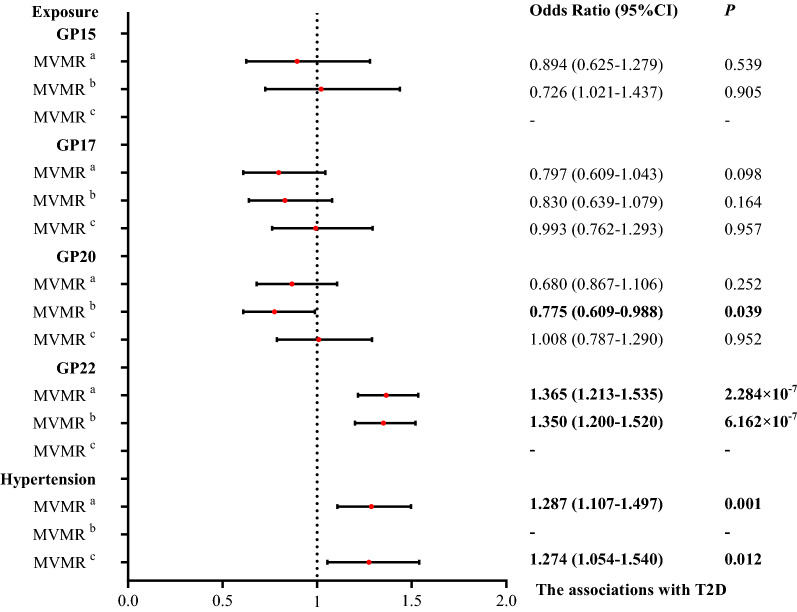
Causal effect estimates on T2D via multivariable Mendelian randomization. The results of significant IgG N-glycans and T2D with hypertension are marked “a” in the top right corner, while the results of only IgG N-glycans are marked “b”, and the results for removing overlapping IgG N-glycans are marked “c”. GP: glycan peak; T2D: Type 2 diabete

Likewise, we further performed a multivariable MR to simultaneously estimate the direct effect of hypertension on T2D conditioned on selected related IgG N-glycans in univariable MR (N_SNPs_ = 288). The multivariable MR effect estimates of genetically proxied hypertension was associated with increased odds of T2D (OR = 1.287, 95% CI = 1.107–1.497, *P* = 0.001; Fig. 3, Additional file 5: Table S6). Findings for genetically proxied hypertension on T2D risk were replicated in the other two MR methods (OR = 1.281, 95% CI = 1.101–1.491, *P* = 0.001, Additional file 3: Fig. S3; OR = 1.408, 95% CI = 1.158–1.711,* P* = 5.895 × 10^–4^, Additional file 4: Fig. S4). The sensitivity analyses yielded similar causal associations of T2D and hypertension via univariable and multivariable MR models, as presented in Additional file 5: Tables S8 and S10. The results of the replicated analysis were inconsistent with the primary analysis (OR = 0.960, 95% CI = 0.784–1.174, *P* = 0.690; Additional file 5: Table S16). GP22 remained a strong risk factor in multivariable MR (OR = 1.365, 95% CI = 1.213–1.535; OR = 1.358, 95% CI = 1.207–1.529; OR = 1.462, 95% CI = 1.234–1.732; all *P*s < 0.05). In addition, the estimates of GP17 on T2D were marginally statistically significant in size in the weighted median method (OR = 0.671, 95% CI = 0.458–0.984, *P* = 0.041) but not in the IVW method and MR‒Egger method. We assessed the causal effect of IgG N-glycans on T2D risk using genetic instruments for hypertension-related IgG N-glycans. We found causal evidence for the effects of GP22 on T2D (OR = 1.350, 95% CI = 1.200–1.520,* P* = 6.162 × 10^–7^). The MR‒Egger and weighted median methods reported consistent findings. The results of the IVW method of GP20 and T2D showed a protective effect (OR = 0.755, 95% CI = 0.609–0.988, *P* = 0.039). After adjusting for overlapping IgG N-glycans in multivariable MR analysis, hypertension remained a risk factor by using the IVW method and MR‒Egger method (OR = 1.274, 95% CI = 1.054–1.540, *P* = 0.012; OR = 1.300, 95% CI = 1.073–1.574, *P* = 0.007), but not in the weighted median method (OR = 1.209, 95% CI = 0.921–1.586, *P* = 0.172). All multivariable MR‒Egger intercept analyses did not provide evidence for horizontal pleiotropy (*P* = 0.308, *P* = 0.236, *P* = 0.128). Effect estimates for the other sensitivity analyses can be found in Additional file 5: Tables S8 and S10.

## Discussion

To our knowledge, this is the first study to investigate the causal relationship between T2D and hypertension with a bidirectional univariable and multivariable MR design integrating IgG N-glycosylation-QTLs and GWAS data. The univariable MR analyses showed that type 2 diabetes was associated with higher hypertension risk, and vice versa. The multivariable results, corrected related IgG-glycans, confirmed the bidirectional causal association between T2D and hypertension.

The present study and the bidirectional MR study from the UK biobank consistently demonstrated that T2D had an increased risk on hypertension, which was also consistent in observational studies [7, 35]. However, the reverse relationship from the UK biobank was inconsistent with ours, which has been consistent in previous MR studies showing different causal associations [36, 37]. Our results were supported by recent observational studies. Of note, 10 mmHg in SBP and 5 mmHg had risk effect on incident T2D in the middle-aged and elderly Chinese population [38]. A large random control trial of 14,978 adults without diabetes at baseline provided evidence that higher blood pressure was associated with newly diagnosed diabetes [39]. Our study revealed the bidirectional regulation of T2D and hypertension, which seems biologically plausible. T2D and hypertension are closely interlinked due to common risk factors, including obesity, dyslipidemia, endothelial dysfunction, and atherosclerosis[6, 40, 41]. The shared mechanisms of oxidative stress, inflammation, and activation of the immune system also likely contribute to the relationship between T2D and hypertension [40, 42]. The replication analyses reported inconsistent results in the bidirectional causal link between T2D and SBP. Due to the lack of individual-level data, we could not uniformly correct for confounding factors, which may account for the inconsistency with our results. In addition, differences in the definition of phenotypes may also lead to differences in results. We observed the positive and marginally significant associations in 2SLS regression analyses, which might be due to limited sample size and lack of sufficient statistical power to detect causal associations between T2D and hypertension. More GWAS with a large sample size would be helpful to increase statistical power.

Our study pointed out a non-trivial role of IgG-glycans in the development of assessment between T2D and hypertension. Observational data reported similar patterns of IgG glycosylation in T2D or hypertension, which included decreased galactosylation, sialylation, and fucosylation and increased in GlcNAc structures [15, 43, 44]. Twenty-seven N-glycan traits from IgG subclasses were associated with T2D in the Uyghur population [43]. In addition, a study of Chinese Muslim ethnicity and the Han population reported associations of IgG-glycans with hypertension and type 2 diabetes mellitus comorbidity [45]. A study of European samples developed a score based on significant IgG-GPs, which was associated with higher diabetes risk and was robust against confounder and clinical biomarkers [46]. Similarly, using the 2617 participants of the TwinsUK cohort, the glycan score of hypertension was built and indicated that the IgG glycome changes prior to the development of hypertension [47]. IgG glycosylation has potential advantages and greater performance as a biomarker for disease prediction. It is essential to distinguish effect estimates between T2D and hypertension from those of IgG N-glycans with T2D and hypertension. Multivariable MR, in particular where some of the effects caused by exposure on the outcome may operate through others, is applied to disentangle bidirectional causality between T2D and hypertension. Multivariable MR showed robust results and demonstrated that T2D caused higher risk of hypertension, and vice versa. Of note, several causal IgG N-glycans for T2D and hypertension were overlapped. The multivariable MR results after removing overlapped IgG N-glycans remain consistent. The causal IgG N-glycosylation overlapping between T2D and hypertension might be involved in the bidirectional regulation and underpin the development of these comorbidities. Despite significant advances in understanding the pathogenesis and treatments of hypertension, there continues to be debate regarding the pharmacologic treatments of hypertension in diabetic patients [42, 48]. Therefore, understanding the pathological links between T2D and hypertension is a critical component for the comprehensive clinical management of disease prevention. Future studies should focus on the functional network to propose mechanisms of the regulation of IgG N-glycosylation on T2D and hypertension.

Integrating IgG N-glycosylation-QTL and GWAS data provide an opportunity in the causal reference of IgG N-glycosylation on T2D and hypertension through MR methods. The comprehensive IgG N-glycan-QTL resources provided by our study reveal a new richness of detail regarding genetic effects on IgG N-glycosylation patterns and characterize the relationship of IgG N-glycosylation with T2D as well as hypertension. IgG N-glycosylation provides information that can possibly bridge a GWAS gap regarding disease-related SNPs. IgG N-glycans were strongly associated with genetic loci, which might explain additional phenotypic variation in T2D and hypertension in addition to SNP discoveries from GWAS to pathogenic molecular.

This study has several limitations. The cross-sectional nature of our data limits definitive causal inference. The results from MR analyses utilizing genetically predicted IgG N-glycosylation, T2D, and hypertension do not prove causation but provide supportive evidence. Although “multi-omics” data and phenotypic data are measured in the same population to control confounding factors, it was limited by the small sample size. The number of SNPs used for assessing the causal association between T2D and hypertension was another concern for sample size, which also resulted in a small proportion of the genetic variance explained. Further studies with large sample size are required greatly on the proportion of variance in the risk factor explained by the IVs. Our findings might be affected by the disequilibrium of sex (31.53% for males vs. 68.47% for females) and relatively low percentage of hypertensive individuals for their age because the relative abundance of IgG N-glycans might vary by the sex differences and differ in participants with hypertension. In addition, the present MR analyses conducted in participants of Chinese descent might limit the generalization of our findings to other ancestry groups. In addition to the above points, statistical power to detect potentially causal relationships through our MR studies was limited for some traits, at least for smaller effects, including some of those observed in our traditional epidemiological analyses.

In summary, T2D was associated with higher hypertension risk, and vice versa, performing bidirectional regulation through IgG N-glycosylation. Evaluation of the genetic and IgG N-glycosylation overlap between T2D and hypertension can be beneficial to understand the shared biological mechanisms underlying this comorbidity. Future studies are needed to comprehensively characterize the mechanisms of IgG N-glycosylation, which is involved in T2D and hypertension.

## Supplementary Information

**Additional file 1: Figure S1.** Causal effect estimates on hypertension via Multivariable Mendelian randomization using the MR-Egger method. The results of significant IgG N-glycans and T2D with hypertension are marked “a” in the top right corner, while the results of only IgG N-glycans are marked “b”, and the results for removing overlapping IgG N-glycans (GP15 and GP22) are marked “c”. CI: confidence intervals; GP: glycan peak; MVMR: MVMR: Multivariable Mendelian Randomization; OR: odds ratio; T2D: Type 2 diabetes.

**Additional file 2: Figure S2.** Causal effect estimates on hypertension via Multivariable Mendelian randomization using the weighted median method. The results of significant IgG N-glycans and T2D with hypertension are marked “a” in the top right corner, while the results of only IgG N-glycans are marked “b”, and the results for removing overlapping IgG N-glycans (GP15 and GP22)are marked “c”. CI: confidence intervals; GP: glycan peak; MVMR: MVMR: Multivariable Mendelian Randomization; OR: odds ratio; T2D: Type 2 diabetes.

**Additional file 3: Figure S3.** Causal effect estimates on T2D via multivariable Mendelian randomization using the MR-Egger method. The results of significant IgG N-glycans and T2D with hypertension are marked “a” in the top right corner, while the results of only IgG N-glycans are marked “b”, and the results for removing overlapping IgG N-glycans (GP15 and GP22)are marked “c”. CI: confidence intervals; GP: glycan peak; MVMR: MVMR: Multivariable Mendelian Randomization; OR: odds ratio; T2D: Type 2 diabetes.

**Additional file 4: Figure S4.** Causal effect estimates on T2D via multivariable Mendelian randomization using the weighted median method. The results of significant IgG N-glycans and T2D with hypertension are marked “a” in the top right corner, while the results of only IgG N-glycans are marked “b”, and the results for removing overlapping IgG N-glycans (GP15 and GP22)are marked “c”. CI: confidence intervals; GP: glycan peak; MVMR: MVMR: Multivariable Mendelian Randomization; OR: odds ratio; T2D: Type 2 diabetes.

**Additional file 5.** Additional tables.

## Data Availability

All data generated or analyzed during this study are included in this published article and its additional information files.
