# Supplementary material for: Leveraging IgG N-glycosylation to infer the causality between T2D and hypertension
Source: Diabetol Metab Syndr. 2023 Apr 25;15:80. doi: 10.1186/s13098-023-01053-6 (PMC10127371; doi:10.1186/s13098-023-01053-6)
Supplement: Supplementary file 1 — Additional file 1: Figure S1. Causal effect estimates on hypertension via Multivariable Mendelian randomization using the MR-Egger method. The results of significant IgG N-glycans and T2D with hypertension are marked “a” in the top right corner, while the results of only IgG N-glycans are marked “b”, and the results for removing overlapping IgG N-glycans (GP15 and GP22) are marked “c”. CI: confidence intervals; GP: glycan peak; MVMR: MVMR: Multivariable Mendelian Randomization; OR: odds ratio; T2D: Type 2 diabetes. [file 13098_2023_1053_MOESM1_ESM.pdf]

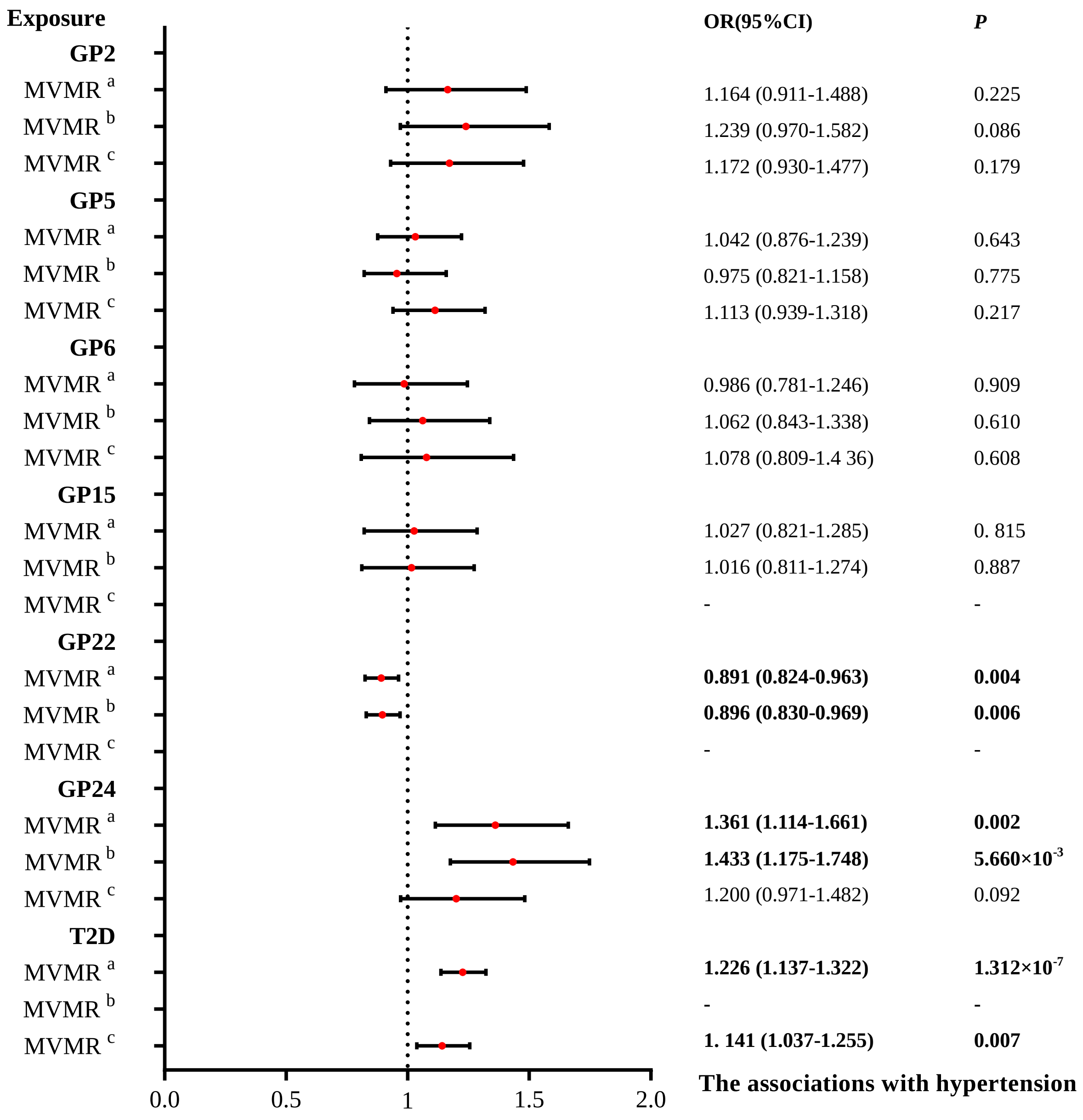

Exposure
OR(95%CI)
P
GP2
MVMR a 1.164 (0.911-1.488) 0.225
MVMR b 1.239 (0.970-1.582) 0.086
MVMR c 1.172 (0.930-1.477) 0.179
GP5
MVMR a 1.042 (0.876-1.239) 0.643
MVMR b 0.975 (0.821-1.158) 0.775
MVMR c 1.113 (0.939-1.318) 0.217
GP6
MVMR a 0.986 (0.781-1.246) 0.909
MVMR b 1.062 (0.843-1.338) 0.610
MVMR c 1.078 (0.809-1.4 36) 0.608
GP15
MVMR a 1.027 (0.821-1.285) 0. 815
MVMR b 1.016 (0.811-1.274) 0.887
MVMR c - -
GP22
MVMR a 0.891 (0.824-0.963) 0.004
MVMR b 0.896 (0.830-0.969) 0.006
MVMR c - -
GP24
MVMR a 1.361 (1.114-1.661) 0.002
MVMR b 1.433 (1.175-1.748) 5.660×10-3
MVMR c 1.200 (0.971-1.482) 0.092
T2D
MVMR a 1.226 (1.137-1.322) 1.312×10-7
MVMR b - -
MVMR c 1. 141 (1.037-1.255) 0.007
0.0
0.5
1
1.5
2.0
The associations with hypertension
