# Supplementary material for: Leveraging IgG N-glycosylation to infer the causality between T2D and hypertension
Source: Diabetol Metab Syndr. 2023 Apr 25;15:80. doi: 10.1186/s13098-023-01053-6 (PMC10127371; doi:10.1186/s13098-023-01053-6)
Supplement: Supplementary file 2 — Additional file 2: Figure S2. Causal effect estimates on hypertension via Multivariable Mendelian randomization using the weighted median method. The results of significant IgG N-glycans and T2D with hypertension are marked “a” in the top right corner, while the results of only IgG N-glycans are marked “b”, and the results for removing overlapping IgG N-glycans (GP15 and GP22)are marked “c”. CI: confidence intervals; GP: glycan peak; MVMR: MVMR: Multivariable Mendelian Randomization; OR: odds ratio; T2D: Type 2 diabetes. [file 13098_2023_1053_MOESM2_ESM.pdf]

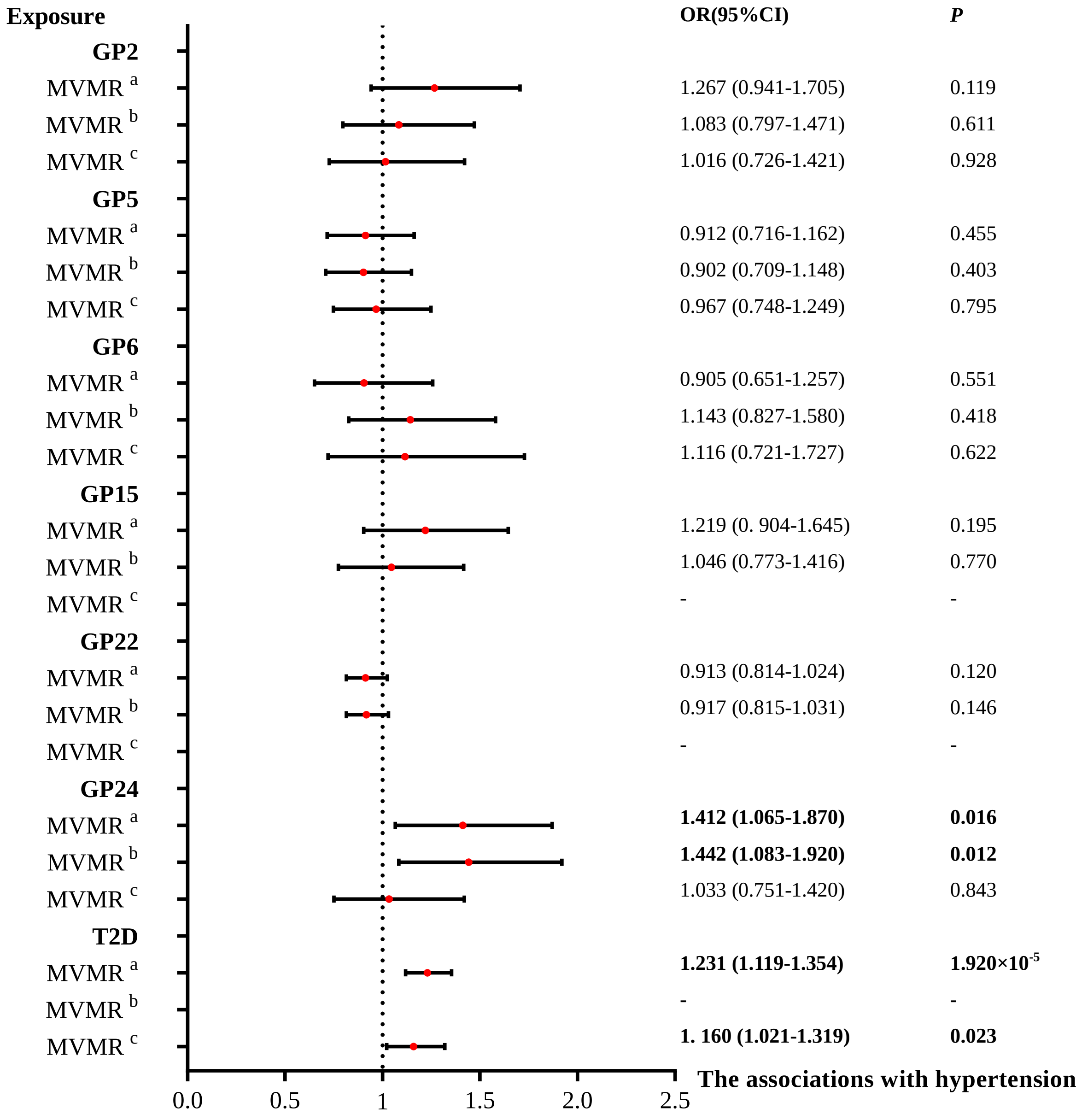
Exposure
OR(95%CI)
P
GP2
MVMR a 1.267 (0.941-1.705) 0.119
MVMR b 1.083 (0.797-1.471) 0.611
MVMR c 1.016 (0.726-1.421) 0.928
GP5
MVMR a 0.912 (0.716-1.162) 0.455
MVMR b 0.902 (0.709-1.148) 0.403
MVMR c 0.967 (0.748-1.249) 0.795
GP6
MVMR a 0.905 (0.651-1.257) 0.551
MVMR b 1.143 (0.827-1.580) 0.418
MVMR c 1.116 (0.721-1.727) 0.622
GP15
MVMR a 1.219 (0. 904-1.645) 0.195
MVMR b 1.046 (0.773-1.416) 0.770
MVMR c - -
GP22
MVMR a 0.913 (0.814-1.024) 0.120
MVMR b 0.917 (0.815-1.031) 0.146
MVMR c - -
GP24
MVMR a 1.412 (1.065-1.870) 0.016
MVMR b 1.442 (1.083-1.920) 0.012
MVMR c 1.033 (0.751-1.420) 0.843
T2D
MVMR a 1.231 (1.119-1.354) 1.920×10-5
MVMR b - -
MVMR c 1. 160 (1.021-1.319) 0.023
0.0
0.5
1
1.5
2.0
2.5
The associations with hypertension
