# Supplementary figures and images for: Leveraging IgG N-glycosylation to infer the causality between T2D and hypertension
Source: Diabetol Metab Syndr. 2023 Apr 25;15:80. doi: 10.1186/s13098-023-01053-6 (PMC10127371; doi:10.1186/s13098-023-01053-6)

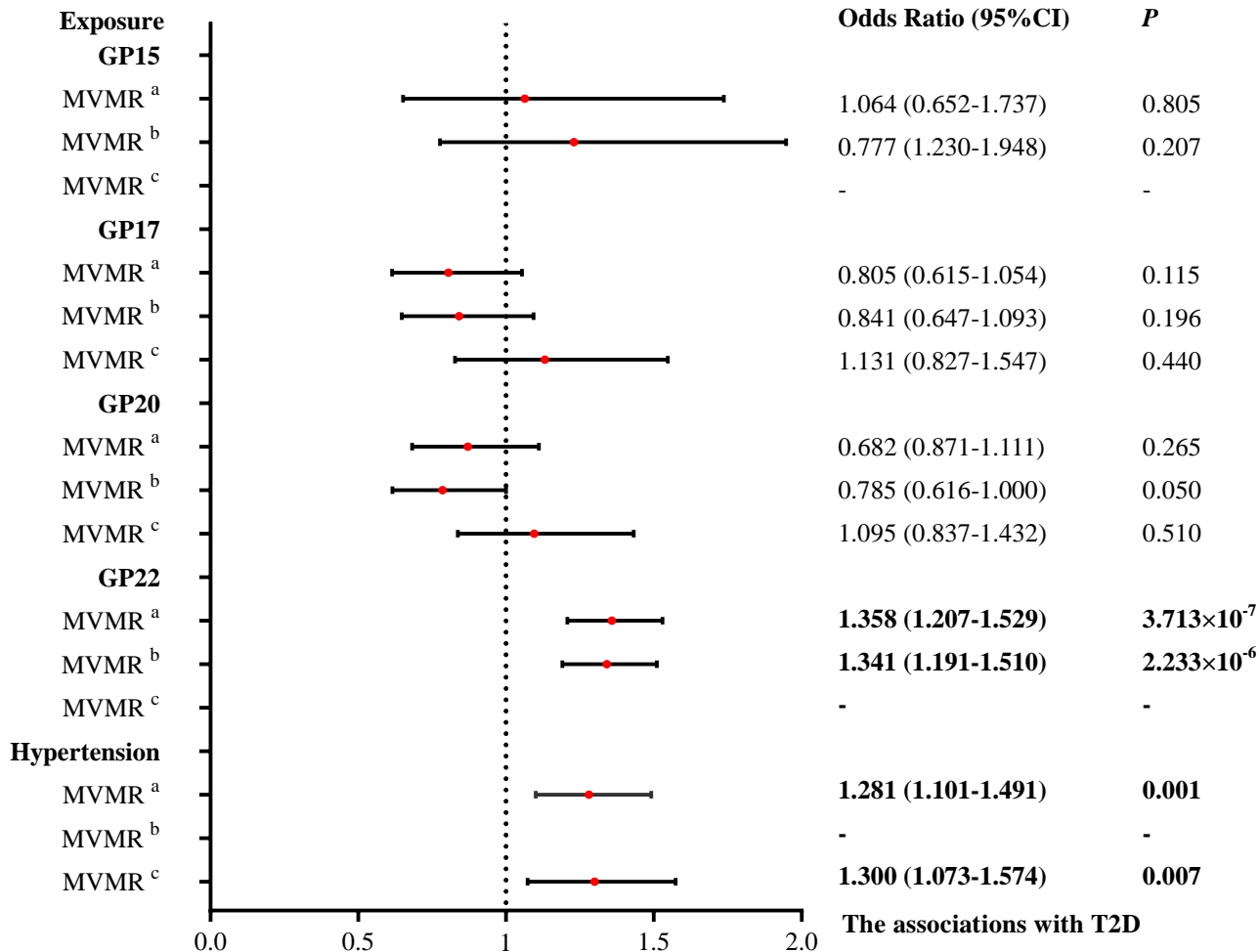

Supplement: Supplementary file 3 — Additional file 3: Figure S3. Causal effect estimates on T2D via multivariable Mendelian randomization using the MR-Egger method. The results of significant IgG N-glycans and T2D with hypertension are marked “a” in the top right corner, while the results of only IgG N-glycans are marked “b”, and the results for removing overlapping IgG N-glycans (GP15 and GP22)are marked “c”. CI: confidence intervals; GP: glycan peak; MVMR: MVMR: Multivariable Mendelian Randomization; OR: odds ratio; T2D: Type 2 diabetes. [file 13098_2023_1053_MOESM3_ESM.pdf]

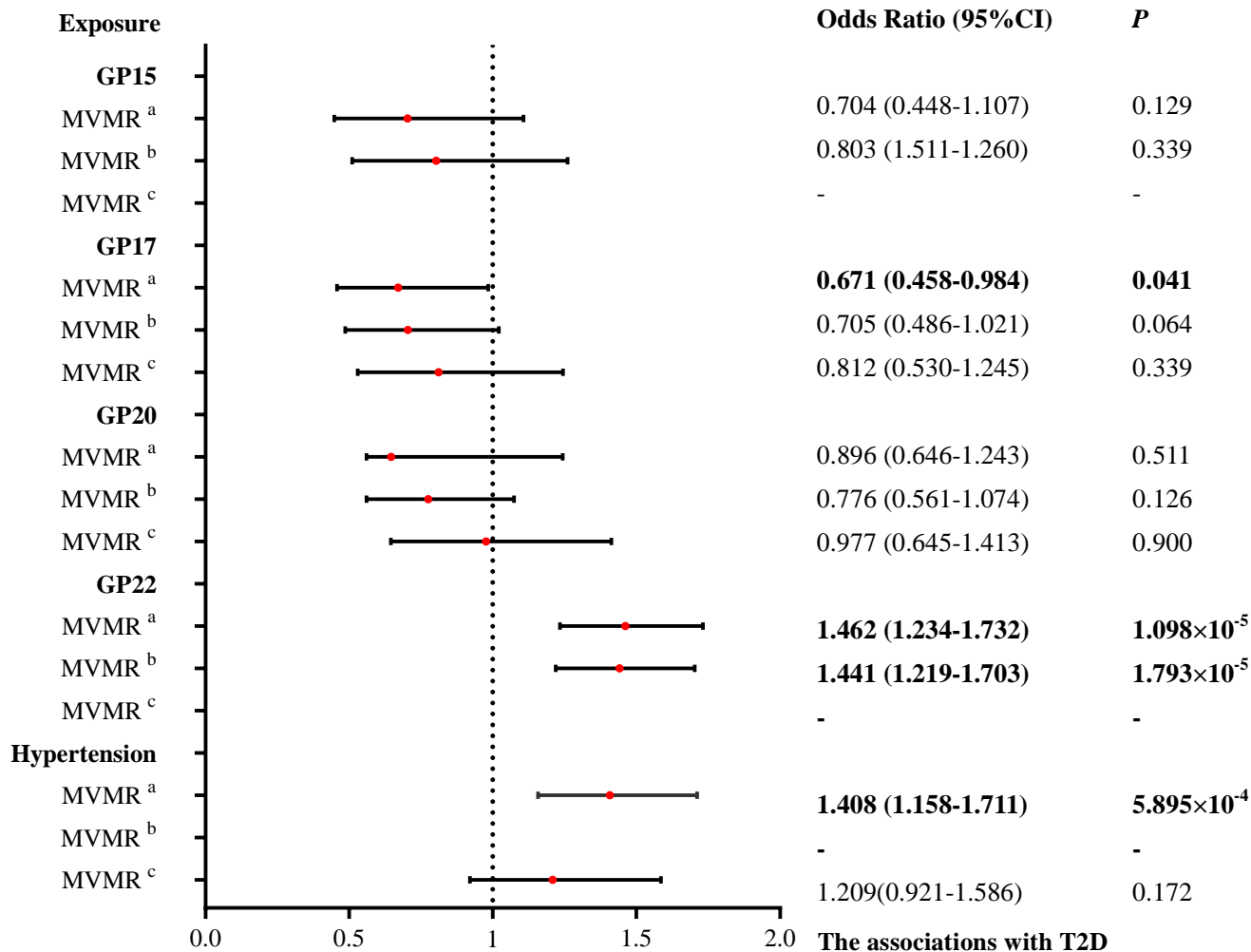

Supplement: Supplementary file 4 — Additional file 4: Figure S4. Causal effect estimates on T2D via multivariable Mendelian randomization using the weighted median method. The results of significant IgG N-glycans and T2D with hypertension are marked “a” in the top right corner, while the results of only IgG N-glycans are marked “b”, and the results for removing overlapping IgG N-glycans (GP15 and GP22)are marked “c”. CI: confidence intervals; GP: glycan peak; MVMR: MVMR: Multivariable Mendelian Randomization; OR: odds ratio; T2D: Type 2 diabetes. [file 13098_2023_1053_MOESM4_ESM.pdf]
